# Supplementary material for: Weather Factors Associated with Reduced Risk of Dengue Transmission in an Urbanized Tropical City
Source: Int J Environ Res Public Health. 2021 Dec 29;19(1):339. doi: 10.3390/ijerph19010339 (PMC8751148; doi:10.3390/ijerph19010339)
Supplement: Supplementary file 1 [file ijerph-19-00339-s001.zip › ijerph-1420816-supplementary.pdf]

Table S1: Epidemiological characteristics of dengue cases in Singapore from 2012-2019

	Host					Virus				Vector	
Year	Number of cases	Incidence per 100,000	Number of imported cases	Total deaths	Case-fatality rate	Serotype 1 (%)	Serotype 2 (%)	Serotype 3 (%)	Serotype 4 (%)	Aedes House Index (%)	Clusters as major breeding sites (10 or more cases)
2012	4,632	82.2	263	2	0.0	19.4	74.1	5.7	0.8	0.28	328 (21)
2013	22,170	404.9	307	8	0.1	61.8	24.6	11.6	2.0	0.30	1,475 (188)
2014	18,326	325.6	514	6	0.1	79.4	18.0	2.5	0.2	0.26	1,418 (137)
2015	11,294	196.1	438	6	0.1	43.2	44.5	11.2	1.1	1.24	1,114 (108)
2016	13,085	229.1	195	12	0.2	29.0	51.1	18.4	1.5	1.2	1,432 (104)
2017	2,767	44.9	249	2	0.04	24.1	45.1	21.3	9.5	0.58	197 (11)
2018*	3,283	54.4	215	5	0.1	NR	NR	NR	NR	0.96	243 (29)
2019#	16,100	282.3	NR	20	0.12	NR	NR	NR	NR	NR	NR

*NR figures were not reported due to a change in the Ministry of Health's annual communicable diseases report

#NR figures as the 2019 communicable disease report has not been published at the time of writing this paper. Incidence per 100,000 calculated using population figures from Singapore's Department of Statistics

Table S2: Estimated parameters of distributed lag non-linear model

**Meteorological Factor**	**QAIC**	**Maximum Lagged Number of Weeks**	δ_1	δ_2
PSI	4955.54	16	4	4
Total Rainfall	4969.682	15	6	3
Mean Temperature	5109.439	16	2	5
Max Temperature	5100.402	16	2	5
Min Temperature	5141.385	16	2	5
Wind Speed	4996.581	5	4	5

Table S3: Predictive model performance using 2012-2018 data as training data and 2019 data as test data

	MAE	RMSE
Base model	46.1	57.3
Base model with PSI	44.8	51.5
Base model with Rainfall	43.7	59.6
Base model with Mean temperature	43.5	52.3
Base model with Max temperature	41.4	55.9
Base model with Min temperature	44.1	50.8

Cross Correlation Graph

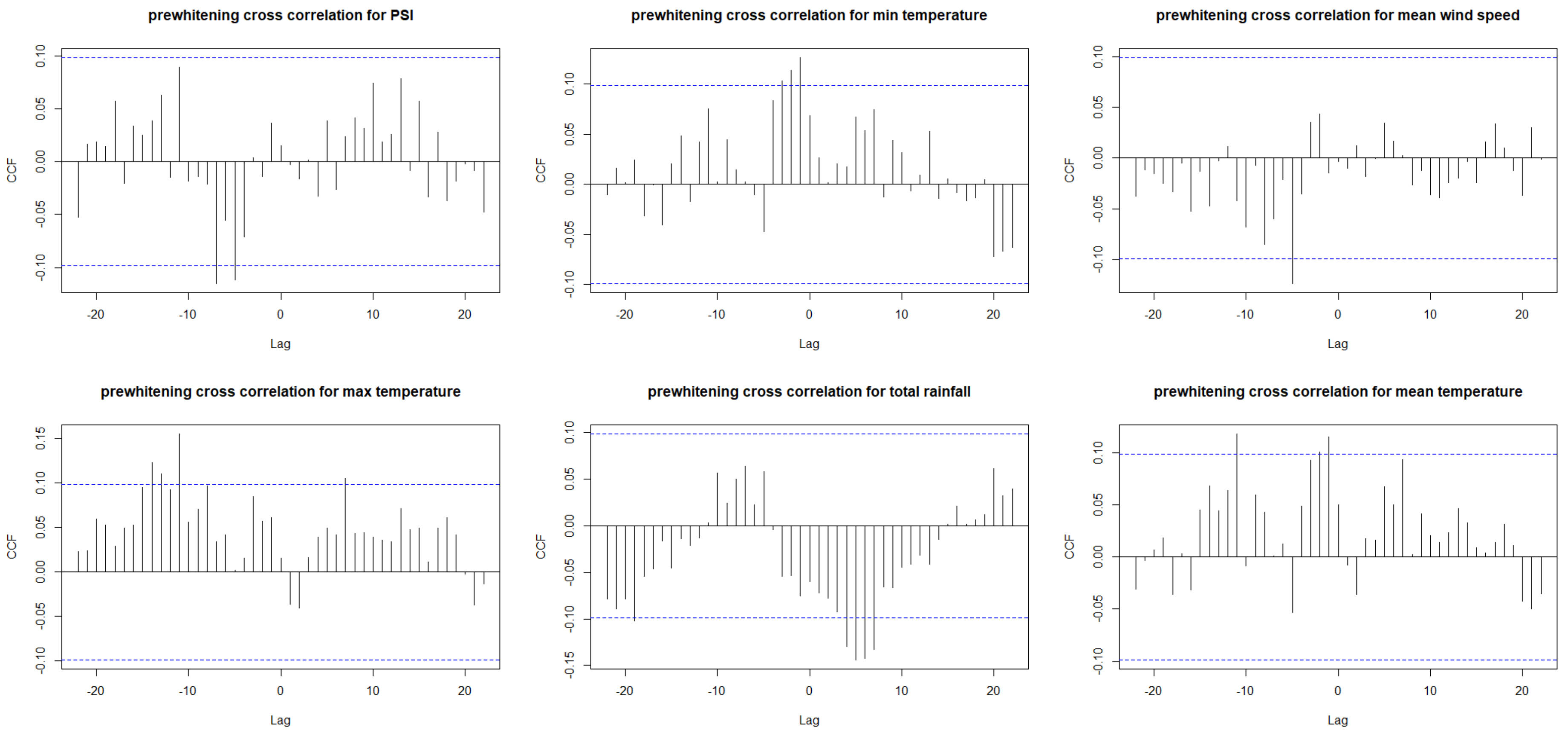

Figure S1: Prewhitening cross correlation graphs between six weather factors and dengue cases

ARIMA Model

With an obvious trend detected in the time series data, differencing technique was conducted to make the time series stationary and stabilize the variance using logarithm transformation. Autocorrelation function graph and partial autocorrelation function suggested significant autocorrelation at lag 1 (Figure S1). Hence, the final model turns to be ARIMA (1, 1, 0). Residual diagnostics was conducted through Figure S2. P-value of 'Ljung-Box' test at significance level 0.05 with 10 lags was 0.3686.

However, some significant signals appeared at lag 43 and lag 49 (Figure S1). These spikes might imply some seasonal patterns of dengue. From Figure 1, peaks were observed around mid-year period during 2012-2015, 2019. In 2016, peak appeared at start of year. Although seasonal patterns were apparent, the seasonal period was not strictly 52 weeks (one year). SARIMA $(0,1,1)(0,1,1)_{52}$ was tried but Ljung-Box test was not passed after lag 43 (Figure S2). Thus seasonal patterns in this weekly data should be captured by a more sophisticated model.

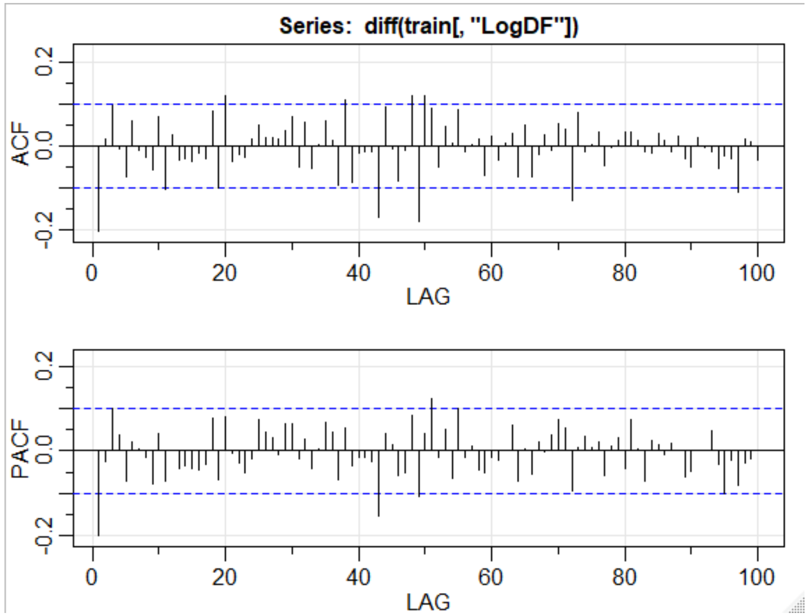

Figure S2: Acf & Pacf graphs of differenced time series of weekly dengue cases with logarithm transformation

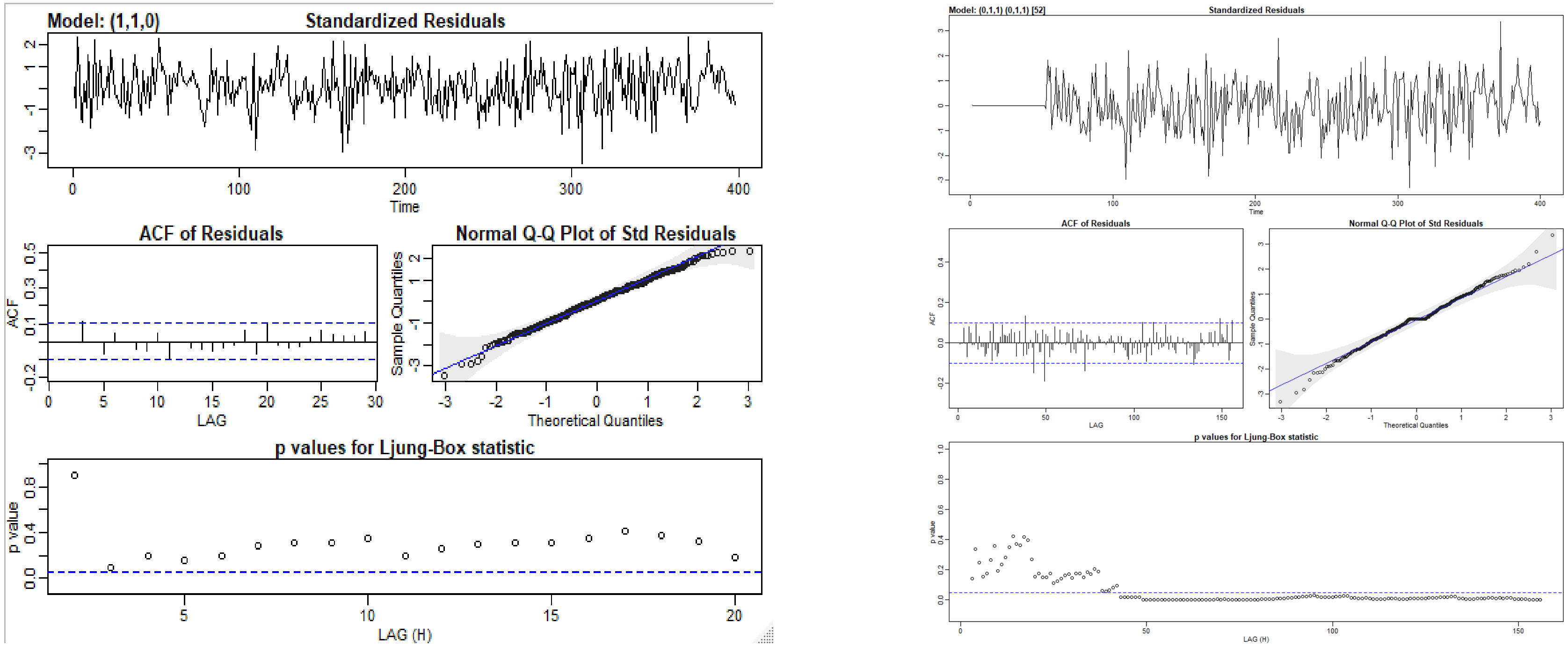

Figure S3: Residual plots and Ljung-Box tests (Residual Diagnostics): Left panel is ARIMA(1, 1, 0) and right panel is SARIMA $(0, 1, 1)(0, 1, 1)_{52}$

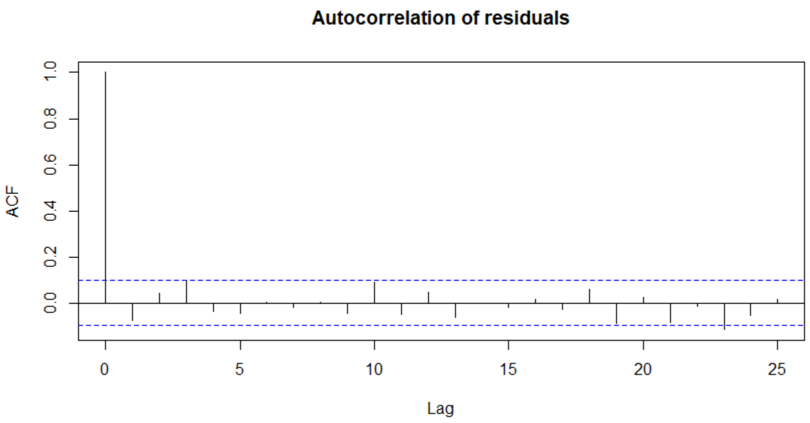

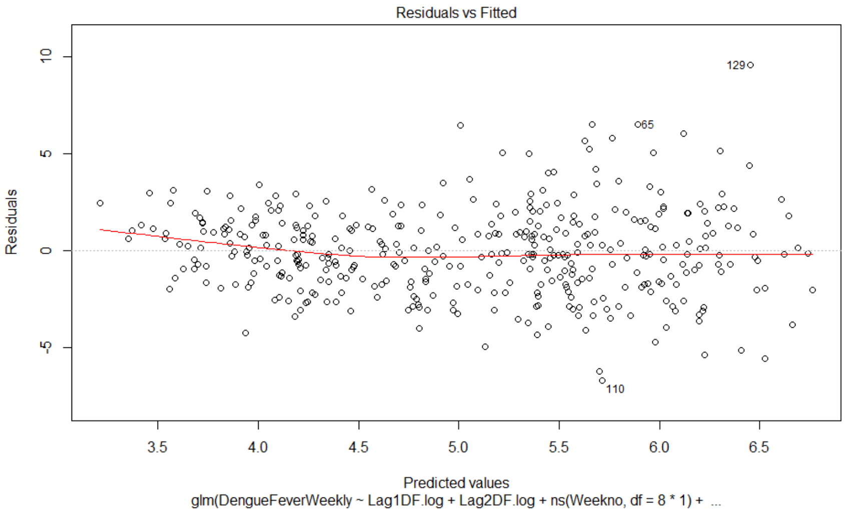

Figure S4: Diagnosis of the residual autocorrelation and overdispersion

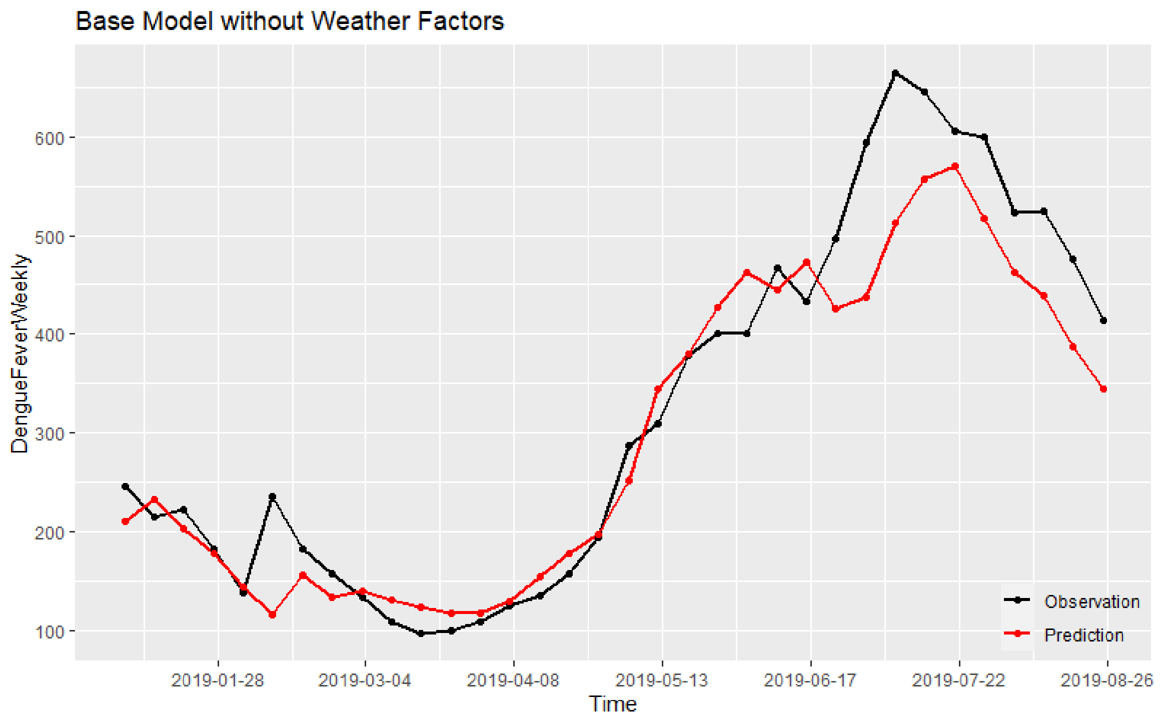

Figure S5: Prediction model of dengue incidence without weather factors

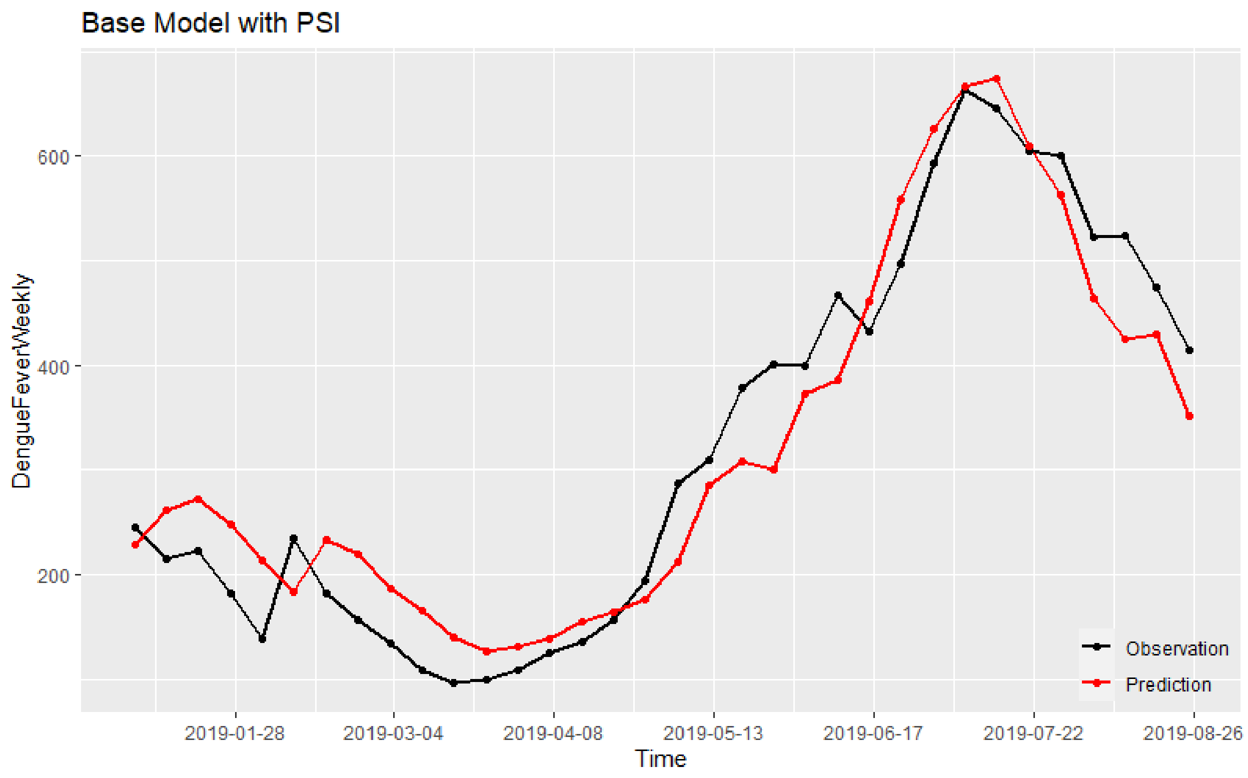

Figure S6: Prediction model of dengue incidence with PSI only

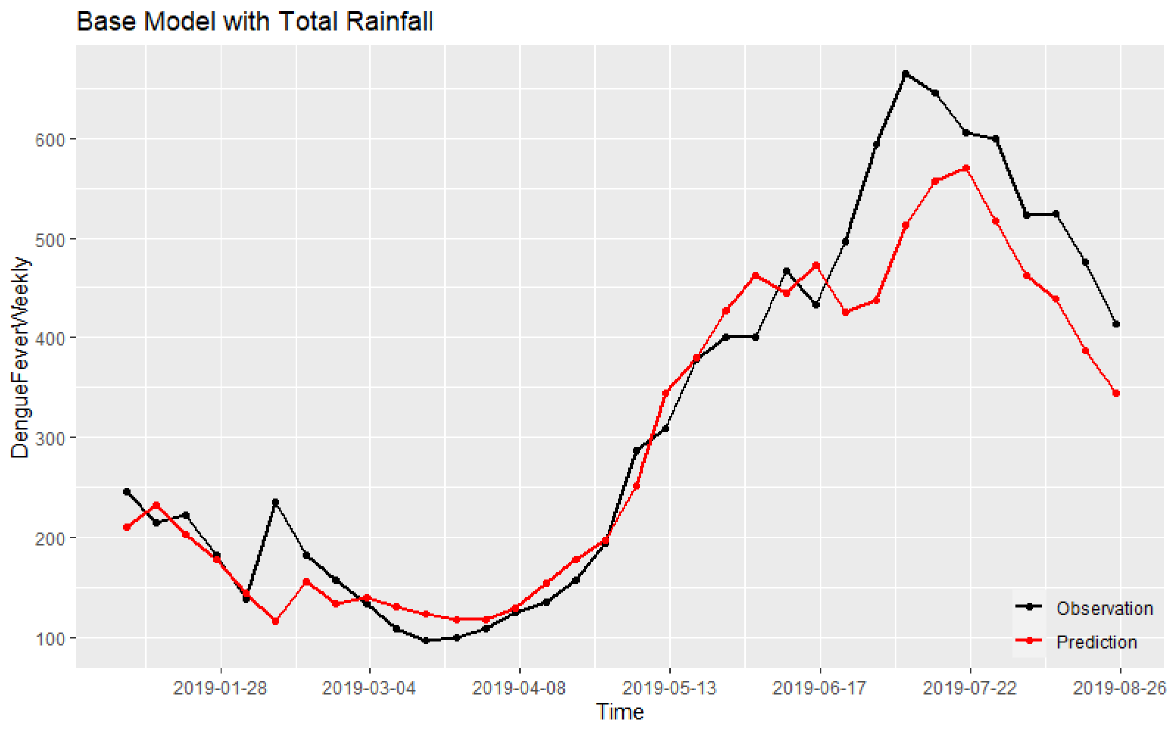

Figure S7: Prediction model of dengue incidence with rainfall only

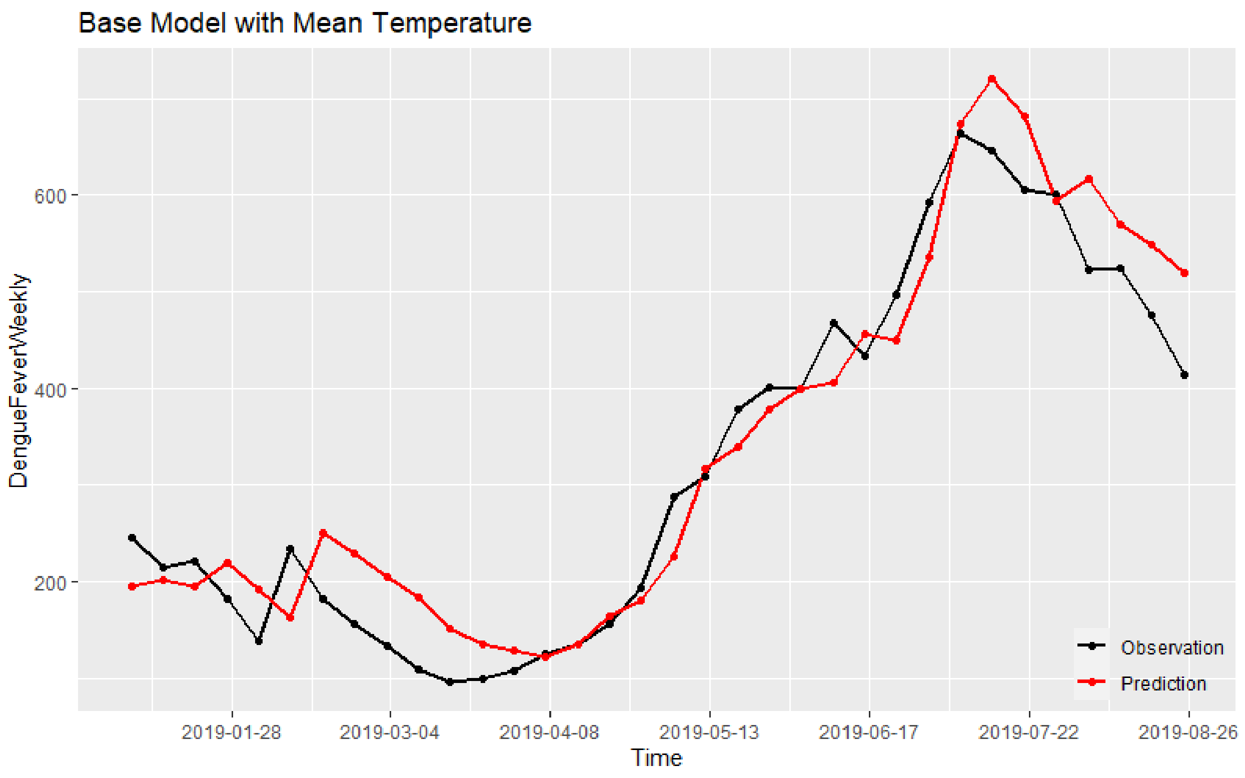

Figure S8: Prediction model of dengue incidence with mean temperature only

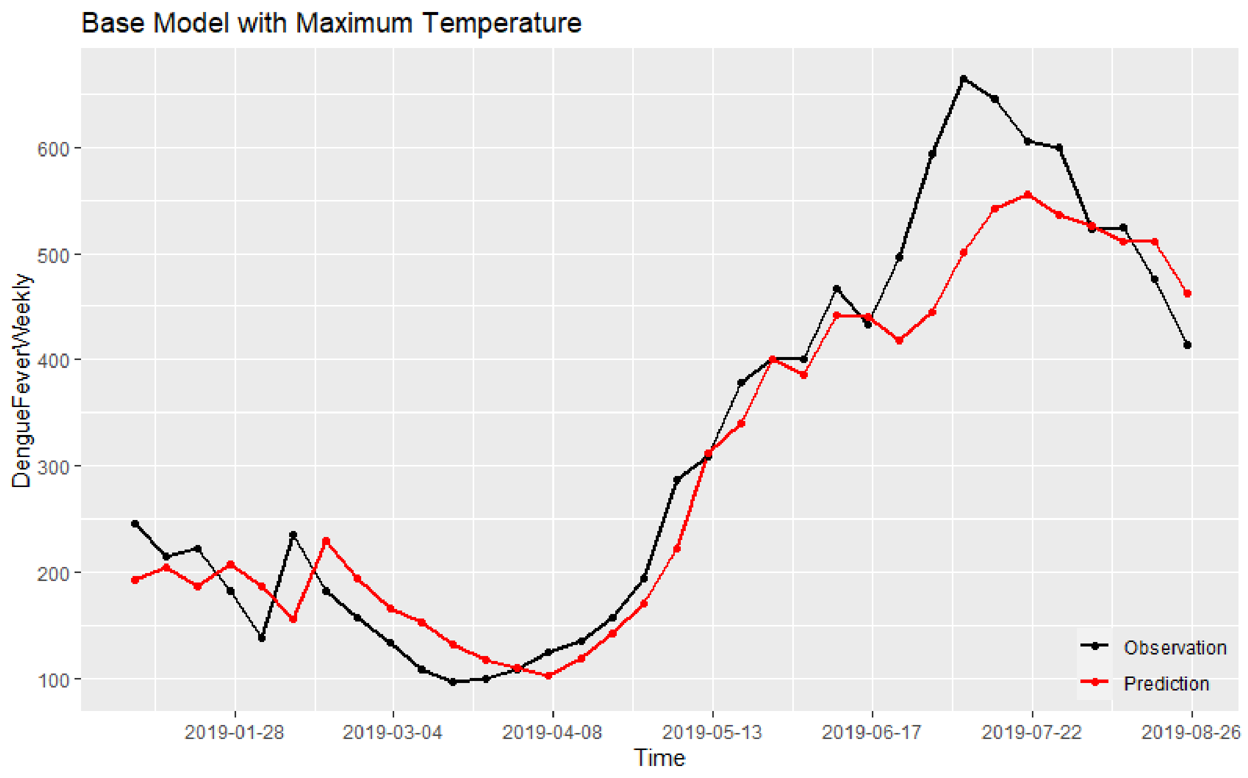

Figure S9: Prediction model of dengue incidence with maximum temperature only

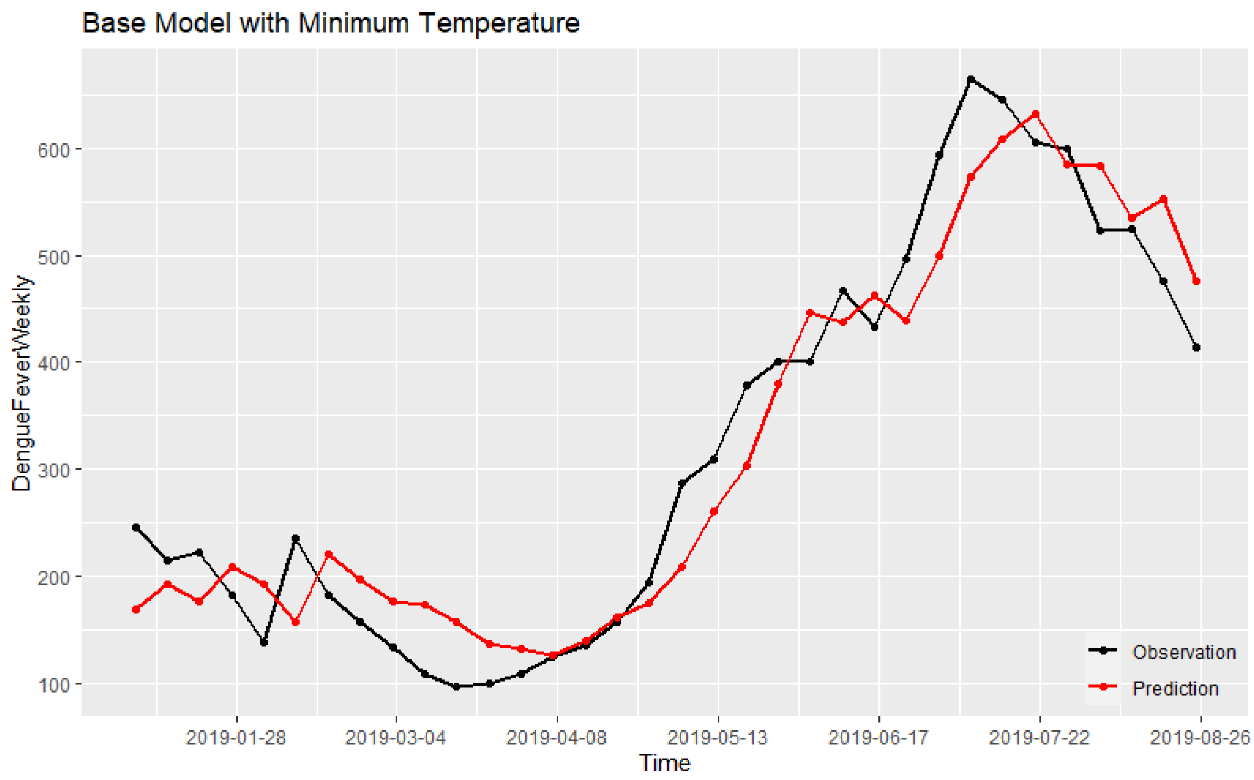

Figure S10: Prediction model of dengue incidence with minimum temperature only

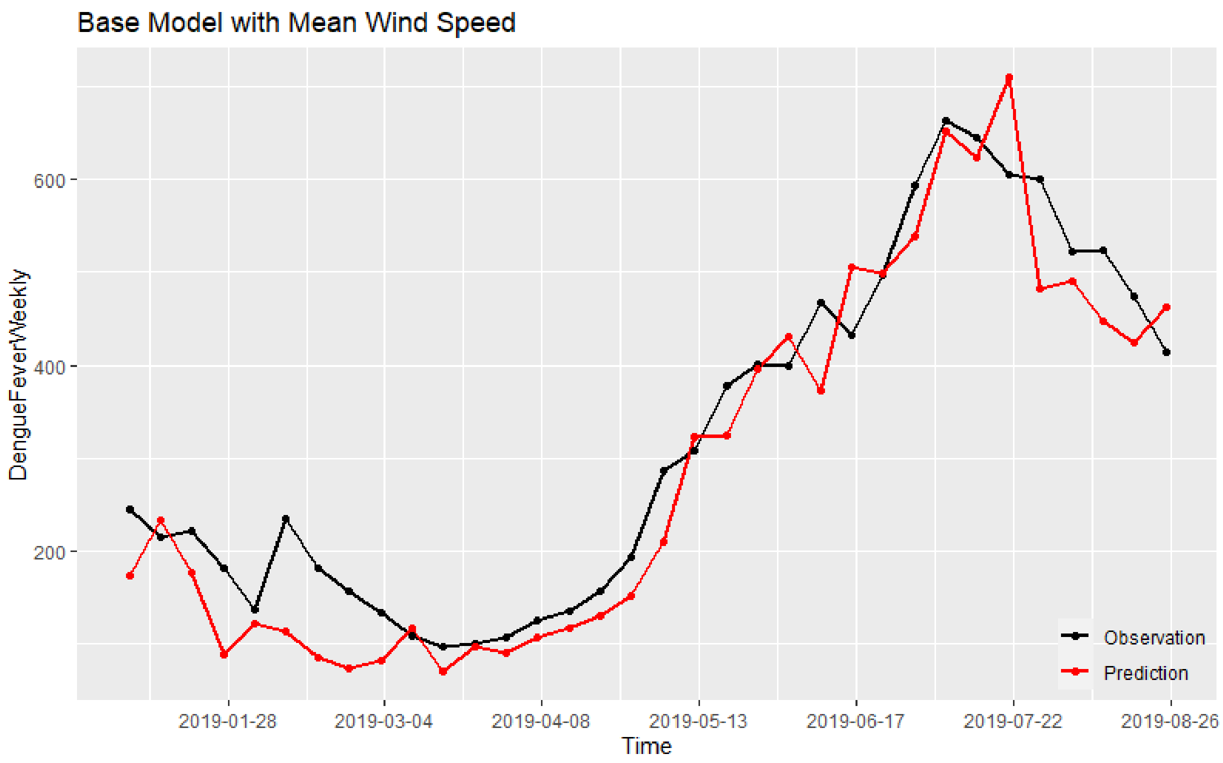

Figure S11: Prediction model of dengue incidence with wind speed only

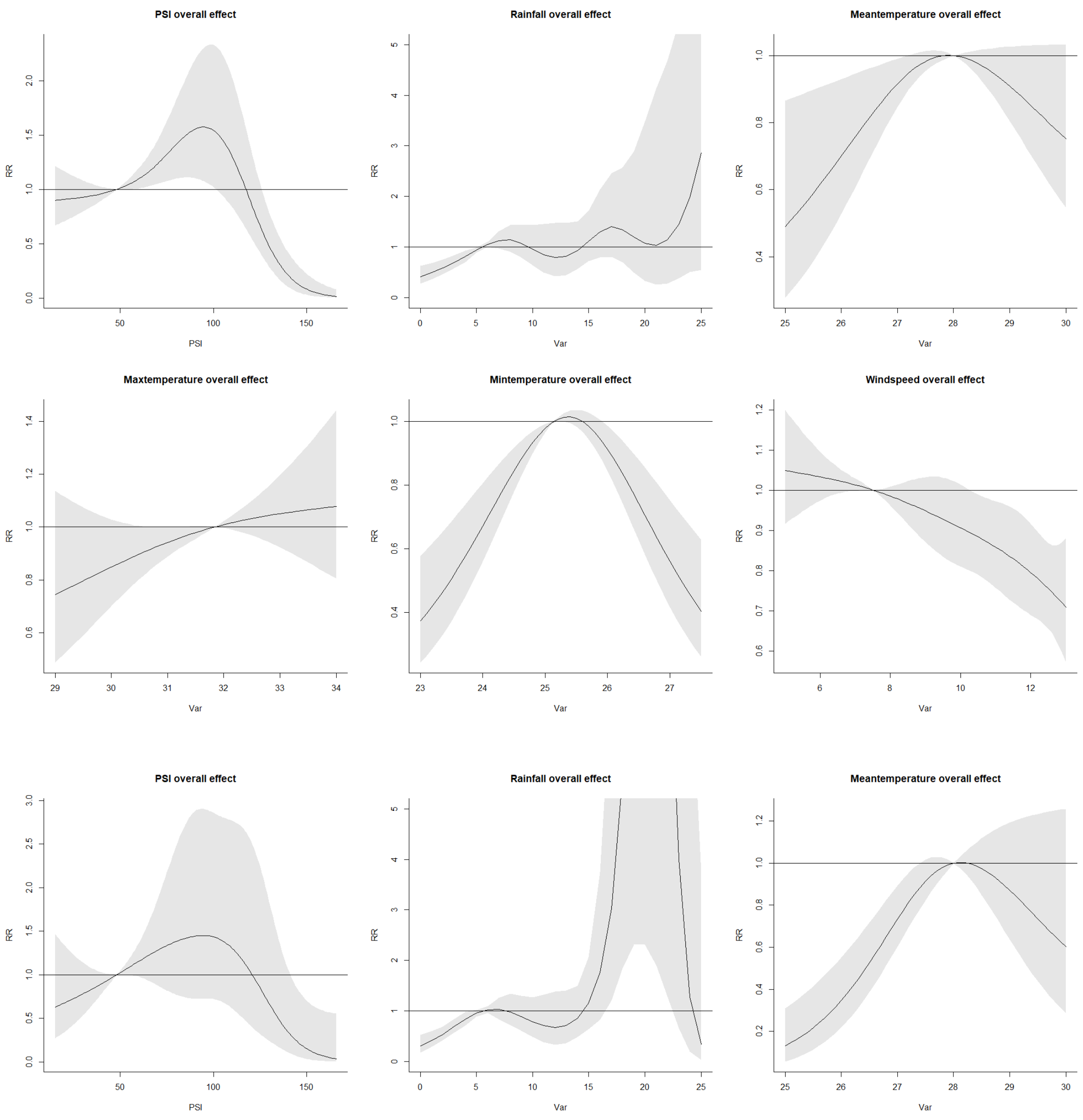
PSI overall effect
RR
PSI
Rainfall overall effect
RR
Var
Meantemperature overall effect
RR
Var
Maxtemperature overall effect
RR
Var
Mintemperature overall effect
RR
Var
Windspeed overall effect
RR
Var
PSI overall effect
RR
PSI
Rainfall overall effect
RR
Var
Meantemperature overall effect
RR
Var

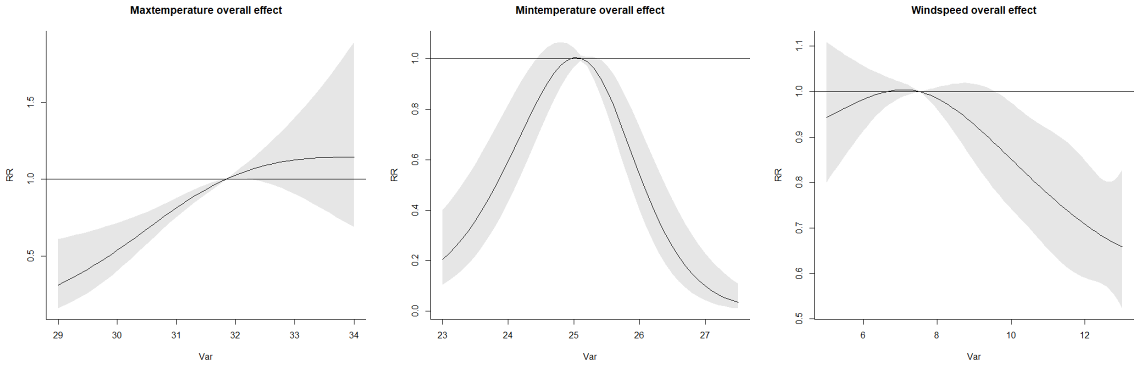

Figure S12 (a): Overall effect with variation in δ. 2df per year (b): Overall effect with variation in δ. 3df per year

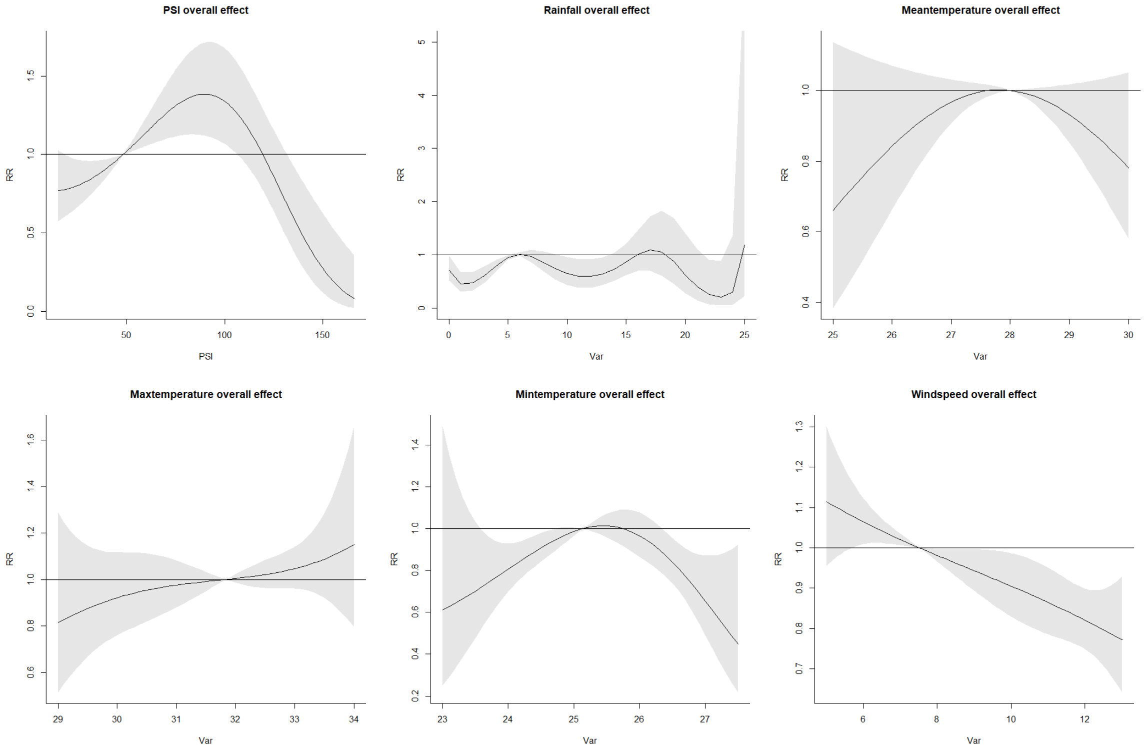

Figure S13: Overall effect of 6 weather variables using polynomial functions
